# Association of Methamphetamine and Opioid Use With Nonfatal Overdose in Rural Communities

**DOI:** 10.1001/jamanetworkopen.2022.26544

**Published:** 2022-08-15

**Authors:** P. Todd Korthuis, Ryan R. Cook, Canyon A. Foot, Gillian Leichtling, Judith I. Tsui, Thomas J. Stopka, Judith Leahy, Wiley D. Jenkins, Robin Baker, Brian Chan, Heidi M. Crane, Hannah L. Cooper, Judith Feinberg, William A. Zule, Vivian F. Go, Angela T. Estadt, Robin M. Nance, Gordon S. Smith, Ryan P. Westergaard, Brent Van Ham, Randall Brown, April M. Young

**Affiliations:** 1 Section of Addiction Medicine, Department of Medicine, Oregon Health & Science University, Portland; 2 Oregon Health & Science University–Portland State University School of Public Health, Portland; 3 Comagine Health, Portland, Oregon; 4 Section of General Internal Medicine, Department of Medicine, University of Washington, Seattle; 5 Department of Public Health and Community Medicine, Tufts University School of Medicine, Boston, Massachusetts; 6 Oregon Health Authority, Portland; 7 Department of Population Science and Policy, Southern Illinois University School of Medicine, Springfield; 8 Department of Behavioral, Social, and Health Education Sciences, Rollins School of Public Health, Emory University, Atlanta, Georgia; 9 Department of Behavioral Medicine and Psychiatry, West Virginia University School of Medicine, Morgantown; 10 Department of Medicine, Infectious Diseases, West Virginia University School of Medicine, Morgantown; 11 RTI International, Research Triangle Park, North Carolina; 12 Department of Health Behavior, Gillings School of Global Public Health, University of North Carolina–Chapel Hill, Chapel Hill; 13 Division of Epidemiology, College of Public Health, The Ohio State University, Columbus; 14 Department of Epidemiology and Biostatistics, School of Public Health, West Virginia University, Morgantown; 15 Division of Infectious Diseases, Department of Medicine, University of Wisconsin School of Medicine and Public Health, Madison; 16 Department of Family Medicine & Community Health, University of Wisconsin School of Medicine & Public Health, Madison; 17 Department of Epidemiology, University of Kentucky, Lexington; 18 Center on Drug and Alcohol Research, University of Kentucky, Lexington

## Abstract

**Importance:**

Overdoses continue to increase in the US, but the contribution of methamphetamine use is understudied in rural communities.

**Objective:**

To estimate the prevalence of methamphetamine use and its correlates among people who use drugs (PWUD) in rural US communities and to determine whether methamphetamine use is associated with increased nonfatal overdoses.

**Design, Setting, and Participants:**

From January 2018 through March 2020, the National Rural Opioid Initiative conducted cross-sectional surveys of PWUD in rural communities in 10 states (Illinois, Kentucky, New Hampshire, Massachusetts, North Carolina, Ohio, Oregon, Vermont, West Virginia, and Wisconsin). Participants included rural PWUD who reported any past-30-day injection drug use or noninjection opioid use to get high. A modified chain-referral sampling strategy identified seeds who referred others using drugs. Data analysis was performed from May 2021 to January 2022.

**Exposures:**

Use of methamphetamine alone, opioids alone, or both.

**Main Outcomes and Measures:**

Unweighted and weighted prevalence of methamphetamine use, any past-180-day nonfatal overdose, and number of lifetime nonfatal overdoses.

**Results:**

Among the 3048 participants, 1737 (57%) were male, 2576 (85%) were White, and 225 (7.4%) were American Indian; the mean (SD) age was 36 (10) years. Most participants (1878 of 2970 participants with any opioid or methamphetamine use [63%]) reported co-use of methamphetamine and opioids, followed by opioids alone (702 participants [24%]), and methamphetamine alone (390 participants [13%]). The estimated unweighted prevalence of methamphetamine use was 80% (95% CI, 64%-90%), and the estimated weighted prevalence was 79% (95% CI, 57%-91%). Nonfatal overdose was greatest in people using both methamphetamine and opioids (395 of 2854 participants with nonmissing overdose data [22%]) vs opioids alone (99 participants [14%]) or methamphetamine alone (23 participants [6%]). Co-use of methamphetamine and opioids was associated with greater nonfatal overdose compared with opioid use alone (adjusted odds ratio, 1.45; 95% CI, 1.08-1.94; *P* = .01) and methamphetamine use alone (adjusted odds ratio, 3.26; 95% CI, 2.06-5.14; *P* < .001). Those with co-use had a mean (SD) of 2.4 (4.2) (median [IQR], 1 [0-3]) lifetime overdoses compared with 1.7 (3.5) (median [IQR], 0 [0-2]) among those using opioids alone (adjusted rate ratio, 1.20; 95% CI, 1.01-1.43; *P* = .04), and 1.1 (2.9) (median [IQR], 0 [0-1]) among those using methamphetamine alone (adjusted rate ratio, 1.81; 95% CI, 1.45-2.27; *P* < .001). Participants with co-use most often reported having tried and failed to access substance use treatment: 827 participants (44%) for both, 117 participants (30%) for methamphetamine alone, and 252 participants (36%) for opioids alone (χ^2^_2_ = 33.8; *P* < .001). Only 66 participants (17%) using methamphetamine alone had naloxone.

**Conclusions and Relevance:**

These findings suggest that harm reduction and substance use disorder treatment interventions must address both methamphetamine and opioids to decrease overdose in rural communities.

## Key Points

**Question:**

How frequently are methamphetamine and opioid use associated with nonfatal overdose in rural communities?

**Findings:**

In this cross-sectional, multistate study of rural communities, 79% of people using drugs reported past-30-day methamphetamine use; nonfatal overdose was greatest in people using both methamphetamine and opioids (22%) vs opioids alone (14%), or methamphetamine alone (6%). People using both substances reported the least access to treatment; only 17% of those using methamphetamine alone had naloxone.

**Meaning:**

These findings suggest that harm reduction and substance use disorder treatment interventions must address methamphetamine use as well as opioids to decrease overdose in rural communities.

## Teaser

This cross-sectional study estimates the prevalence of methamphetamine use and its correlates among people who use drugs in rural US communities.

## Introduction

The US overdose epidemic continues to accelerate despite extensive attempts to expand treatment for opioid use disorder and overdose prevention services. An estimated 100 306 people died of an overdose between April 2020 and March 2021, the greatest number of overdoses ever in a 12-month period.^1^ Reasons for increased overdose rates are multifactorial and include the increasing dominance of fentanyl in the opioid drug supply and its addition to psychostimulants, increases in psychostimulant use that appear to exacerbate the risk of overdose,^2^ and the COVID-19 pandemic.^3^ The age-adjusted rate of drug overdose deaths involving psychostimulants, including methamphetamine, increased 10-fold from 0.5 per 100 000 people in 2009 to 5.0 per 100 000 people in 2019.^4^ Although both cocaine and methamphetamine combined with opioids increase the risk of overdose,^5^ methamphetamine is associated with an increased risk of overdose, regardless of opioid use.^4^

Methamphetamine use has been endemic in the western US for decades, particularly in rural communities where use is more prevalent than in metropolitan areas.^6,7^ Recent data suggest that methamphetamine use has spread to other regions of the US^6,8,9^ where it is associated with unstable housing, low income, and rural residence.^10^ Nationwide, methamphetamine-related hospitalizations increased 270% between 2007 and 2015, accompanied by marked increases in length of stay, inpatient mortality, and cost.^11^ Concomitant use of methamphetamine and opioids is associated with increased injection drug use,^12^ hepatitis C virus infection, and severe mental illness^10^ that amplify adverse outcomes. One study^9^ of fatal drug overdoses involving methamphetamine found that opioids were involved in 64.7% of cases. People who live in rural communities, where access to medications for opioid use disorder and behavioral health services are often limited,^13,14,15^ may be particularly susceptible to the adverse consequences of methamphetamine use.^16^

Despite the associated harms, little is known about the characteristics of methamphetamine use in rural communities or the interplay between methamphetamine and opioid use in the context of a national overdose crisis. People in rural areas who use drugs also have limited access to treatment and prevention services that mitigate harms, compared with individuals in urban settings.^13,14,15^ Increasing understanding of patterns and consequences of methamphetamine use in rural communities may improve approaches to overdose prevention. The objective of this study was to estimate the prevalence of methamphetamine use and its correlates among people who use drugs in rural US communities participating in the national Rural Opioid Initiative (ROI). We hypothesized that co-use of methamphetamine and opioids would be associated with increased nonfatal overdose.

## Methods

### Study Design and Setting

We conducted a cross-sectional survey of people who use drugs in rural counties with high overdose rates in 8 sites covering 10 states (Illinois, Kentucky, New Hampshire, North Carolina, New England [Massachusetts, New Hampshire, and Vermont], Ohio, Oregon, West Virginia, and Wisconsin) participating in the ROI (authors’ unpublished data). The ROI is a cooperative agreement between participating sites and the National Institute on Drug Abuse, the Substance Abuse and Mental Health Services Administration, the Centers for Disease Control and Prevention, and the Appalachian Regional Commission with the goal of better characterizing the rural opioid epidemic and its consequences. All sites obtained local institutional review board approval for research activities and data sharing within the ROI. This report follows the Strengthening the Reporting of Observational Studies in Epidemiology (STROBE) reporting guideline for observational studies. Participants provided consent before beginning the survey.

### Participants

Individuals were eligible for inclusion if they lived in the study area, reported any past-30-day injection drug use or noninjection opioid use “to get high,” were able to communicate in English, and were at least age 15 years at 2 sites and age 18 years at the remaining sites (eTable 1 in the Supplement). No eligible participants were excluded.

Between January 2018 and March 2020, we recruited participants using a modified chain-referral sampling strategy,^17^ based on Respondent Driven Sampling (RDS).^18^ Sites enrolled between 42 and 279 seeds (ie, individuals who met eligibility criteria and agreed to initiate recruitment chains). Seeds were purposively selected from syringe service programs, other support services, and community outreach to represent the sex and racial and ethnic characteristics of the local population of eligible individuals. Seeds recruited 3 to 6 members of their social network (maximum number varied by study) whom they knew to either inject drugs or use opioid medications for nonmedical purposes, using coded invitation coupons so that RDS chains could be tracked. Referred participants recruited their network peers similarly with the goal of maximizing recruitment chains. Participants received $10 to $20 per successfully enrolled peer and $40 to $60 for completion of study procedures.

### Study Procedures

Participants completed a 35- to 90-minute, centrally developed, harmonized, computer-assisted structured questionnaire, collected by Audio Computer-Assisted Self-Interview at 5 sites,^19^ computer-assisted personal interview at 2 sites (REDCap and QDS), and computer-assisted self-interview at 1 site (Qualtrics).^20^ Data were transferred to the national Data Coordinating Center for data quality review and collation of a national analytic data set. Survey domains included demographics (year of birth, gender, race and ethnicity, and housing status), past-30-day and lifetime substance use (heroin, other opioids, methamphetamine, cocaine or crack, alcohol, and tobacco), injection frequency, treatment access, and overdose history. Race and ethnicity were analyzed in this study because overdose rates and access to drug use treatment often vary by race and ethnicity.

### Measures

Participants were asked to report whether they had ever used methamphetamine (termed as “methamphetamine, crystal meth, or amphetamine” in the survey), and if so, how many of the last 30 days they had used methamphetamine to get high. We defined current methamphetamine use as report of 1 or more days of use in the past 30 days. We defined current opioid use as at least 1 day of reported heroin, fentanyl, opioid painkillers, synthetic opioids, buprenorphine, or methadone to get high within the last 30 days. On the basis of their past-30-day use of methamphetamine and opioids, participants were categorized as using opioids without methamphetamine (opioids alone), methamphetamine without opioids (methamphetamine alone), or use of both substances.

We assessed overdose by asking participants, “Have you ever overdosed? By overdose, I mean if you passed out, turned blue, or stopped breathing from using drugs.”^21^ Participants who reported any prior overdose were asked the total number of lifetime overdoses and the date of their last overdose. We used responses to construct 2 variables: (1) any overdose within the past 180 days, derived from the date of last overdose, and (2) the total number of lifetime overdoses. Because some respondents reported extremely high lifetime values, counts were truncated at the 99th percentile of the variable distribution (25 overdoses).

To assess services access, we asked, “In the past 6 months, did you try to get any of the following treatments but were unable to: buprenorphine maintenance, methadone maintenance, naltrexone shots, buprenorphine shots, outpatient drug treatment, residential or inpatient drug treatment, drug detox?” dichotomized as any unsuccessful attempt to access treatment. We also asked, “Do you currently have naloxone or Narcan with you or at home?” eTable 1 in the Supplement reports survey items used to derive measures for the current analysis.

### Statistical Analyses

We estimated the overall prevalence of methamphetamine use by using a random effects model where the estimate was the average of the site-specific effects. Site-specific prevalence estimates were calculated by stratifying the data set by site with 95% CIs obtained by bootstrapping. Using RDS design, data-participant recruitment trees, and network sizes, we calculated unweighted and weighted prevalence estimates by applying the RDS-II estimator.^22^ The goal of RDS weighting is to approximate a simple random sample of a hard-to-reach (hidden) population, where construction of a sampling frame would be difficult or impossible. Network size at 6 sites was ascertained via the harmonized question, “Please think about the people living in [your area] who are at least 18 years of age and who to your knowledge, have used heroin or prescription opioids (through any route of administration) or have injected any type of drug to get high in the past 30 days. How many of these people do you know personally?” One site estimated network size by counting the number of network members listed in the social network inventory who were perceived to inject drugs or use opioids. When the network size item was missing, it was replaced by the number of people a participant recruited plus the person who recruited the participant (1 if recruited; 0 if a seed). If network size was still 0 after this step, a network size of 1 was used, instead. From these data, RDS-II, or Volz-Heckathorn, weights were calculated using the RDS package in R statistical software version 4.0.5 (R Project for Statistical Computing), except for in West Virginia, which did not collect RDS information. RDS weights were not applied to analyses of associations (ie, regression models examining associations between methamphetamine and overdose), as (1) RDS methods were developed for prevalence estimation and (2) weighting has been shown to increase bias in association research.^23^

RDS weight adjustment relies on certain assumptions, which were not fully met in every site, such as low numbers of seeds and long recruitment chains. Weighted and unweighted estimates differed little except in Kentucky and Ohio, which had longer recruitment chains and fewer nongenerative seeds. A sensitivity analysis excluding seeds and the first 2 recruitment waves yielded similar prevalence estimates (eTable 2 in the Supplement).

We described and compared participant characteristics by substance use groups (ie, methamphetamine and opioids, opioids alone, and methamphetamine alone) using χ^2^ tests. A total of 194 participants were missing overdose information or did not report recent use of either methamphetamine or opioids and were excluded from overdose analyses, leaving 2854 participants in the analytic sample for overdose models. There were no substantial differences in demographic characteristics or drug use behaviors between included and excluded participants. Mixed-effects logistic regression modeled the probability of an overdose within the past 180 days and mixed negative binomial regression modeled counts of total lifetime overdoses. Models were adjusted for age, sex, race, and Hispanic ethnicity, as well as binary indicators of past-30-day benzodiazepine use, cocaine use, binge drinking, daily injection drug use, and past-6-month homelessness. Random intercepts were included for study site and recruitment networks within site. Analyses were conducted in R statistical software version 4.0.5 (R Project for Statistical Computing) using the lme4 and emmeans packages at a 2-sided significance level of *P* < .05. Data analysis was performed from May 2021 to January 2022.

## Results

Table 1 presents unweighted and RDS-weighted estimates of the overall and site-specific prevalence of methamphetamine use. In the weighted model, the overall estimated prevalence of methamphetamine use was 79% (95% CI, 57%-91%), slightly lower than the unweighted estimate of 80% (95% CI, 64%-90%). Methamphetamine prevalence varied among sites. Of the 7 sites, New England was the only 1 in which a majority of participants did not report current methamphetamine use (weighted prevalence, 32%; 95% CI, 28%-36%). Methamphetamine use was most prevalent in Oregon, where more than 95% of participants reported using methamphetamine (weighted prevalence, 98.0%; 95% CI, 95.0%-99.6%).

**Table 1.** Overall and Site-Specific Prevalence of MA Use Among Rural Opioid Initiative Participants

Region and state	Unweighted		Weighted	
	Participants, No. (%)	MA prevalence, % (95% CI)	Participants, No. (%)	MA prevalence, % (95% CI)
Overall	3048 (100)	80 (64-90)	12 556 (100)	79 (57-91)
Illinois	173 (6)	80 (73-86)	425 (3)	78 (68-87)
Kentucky	338 (11)	79 (73-82)	1799 (14)	62 (51-72)
North Carolina	350 (11)	93 (90-95)	1406 (11)	91 (86-96)
New England^a^	589 (19)	34 (31-39)	861 (19)	32 (28-36)
Ohio	258 (8)	80 (76-84)	822 (7)	61 (49-74)
Oregon	174 (6)	97 (94-99)	572 (5)	98 (95-100)
Wisconsin	991 (33)	88 (86-90)	6671 (53)	85 (82-88)
West Virginia	175 (6)	51 (43-57)	NA^b^	NA^b^

Abbreviations: MA, methamphetamine; NA, not applicable.

^a^ Includes Massachusetts, New Hampshire, and Vermont.

^b^ West Virginia did not collect respondent driven sampling data required for calculating weights.

The 3048 participants had a mean (SD) age of 36 (10) years; 1737 (57%) were male, 2576 (85%) were White, 225 (7.4%) were American Indian, 116 (3.8%) were Hispanic or Latino, and 1612 (53%) reported being unhoused in the past 6 months. In the past 30 days, 2268 of 2970 participants (76%) reported using either methamphetamine or opioids (mean [SD], 17 [10] days using methamphetamine), and 2580 (85%) reported using opioids (mean [SD], 21 [11] days using opioids). Among respondents reporting any opioid use in the last 30 days, 2102 (82%) reported using heroin, 1744 (67%) reported using opioid painkillers, and 1122 (43%) reported knowingly using fentanyl over the same period. Additionally, 1739 participants (57%) reported injecting drugs at least once per day in the past month and 517 (18%) reported experiencing an overdose within the past 180 days. The mean (SD) number of lifetime overdoses was 2.05 (3.92) (median [IQR], 0 [0-3] overdoses).

Among respondents reporting any methamphetamine or opioid use, the majority (1878 of 2970 participants [63%]) used both methamphetamine and opioids, 702 participants (24%) used opioids alone, and 390 participants (13%) used methamphetamine alone (Table 2). Some differences across racial groups were observed (χ^2^_6_ = 42.6; *P* < .001) with 175 of 225 American Indian (80%) and 49 of 96 (48%) Black respondents reporting co-use of opioids and methamphetamine. Co-use of methamphetamine and opioids was associated with greater daily injection (1222 respondents [65%] for co-use, 211 respondents [54%] for methamphetamine alone, and 289 respondents [41%] for opioids alone; χ^2^_2_ = 122.6; *P* < .001), past-30-day benzodiazepine use (1064 participants [57%] for co-use, 78 participants [20%] for methamphetamine alone, 283 participants [40%] for opioids alone; χ^2^_2_ = 195.5; *P* < .001), and past-30-day binge drinking (630 participants [34%] for co-use, 97 participants [25%] for methamphetamine alone, 185 participants [26%] for opioids alone; χ^2^_2_ = 19.6; *P* < .001). Past-30-day cocaine use was most common among those who reported opioid use without methamphetamine (369 participants [53%] for opioids alone, 884 participants [47%] for co-use, and 68 participants [17%] for methamphetamine alone). Respondents using both methamphetamine and opioids more frequently reported having tried and failed to access substance use treatment (827 participants [44%] for both, 117 participants [30%] for methamphetamine alone, and 252 participants [36%] for opioids alone; χ^2^_2_ = 33.8; *P* < .001), whereas those using methamphetamine alone were least likely to report having a naloxone kit (66 participants [17%] for methamphetamine alone, 275 [39%] for opioids alone, and 733 [39%] for both; χ^2^_2_ = 71.9; *P* < .001) (Table 2).

**Table 2.** Participant Characteristics by MA and Opioid Use Among Rural Opioid Initiative Participants With Recent Use of MA, Opioids, or Both^a^

Characteristic	Participants, No. (%)				*P* value^b^
	Overall (N = 2970)	MA alone (n = 390)	Opioids alone (n = 702)	Opioids and MA (n = 1878)	
Race					
Black	95 (3.2)	12 (3.1)	37 (5.3)	46 (2.4)	<.001
Native American	220 (7.4)	23 (5.9)	22 (3.1)	175 (9.3)	
Other, multiracial, or refused^c^	148 (5.0)	23 (5.9)	39 (5.6)	86 (4.6)	
White	2507 (84)	332 (85)	604 (86)	1571 (84)	
Hispanic ethnicity	112 (3.8)	9 (2.3)	30 (4.3)	73 (3.9)	.24
Gender					
Male	1699 (57)	230 (59)	385 (55)	1084 (58)	.32
Female	1271 (43)	160 (41)	317 (45)	794 (42)	
Age, mean (SD), y	36 (10)	37 (11)	37 (11)	36 (10)	.008^d^
Homeless in last 30 d	1584 (53)	228 (58)	308 (44)	1048 (56)	<.001
Injects daily	1722 (58)	211 (54)	289 (41)	1222 (65)	<.001
Days using methamphetamine in the past 30 d, mean (SD), No.	17 (10)	17 (10)	NA	16 (10)	.20^d^
Days using opioids in the past 30 d, mean (SD), No.	21 (11)	NA	21 (11)	21 (11)	.12^d^
Binge drinking in the last 30 d	912 (31)	97 (25)	185 (26)	630 (34)	<.001
Cocaine use last 30 d	1321 (44)	68 (17)	369 (53)	884 (47)	<.001
Benzodiazepine use last 30 d	1425 (48)	78 (20)	283 (40)	1064 (57)	<.001
Tried to access treatment but could not in last 6 mo	1196 (40)	117 (30)	252 (36)	827 (44)	<.001
Currently has naloxone overdose rescue kit	1074 (36)	66 (17)	275 (39)	733 (39)	<.001
Ever overdosed	1455 (49)	120 (31)	310 (44)	1025 (55)	<.001
Overdosed in the past 180 d	532 (18)	24 (6)	99 (14)	409 (22)	<.001
Total lifetime overdoses, No.					
Mean (SD)	2.05 (3.94)	1.07 (2.85)	1.70 (3.52)	2.39 (4.23)	<.001^b^
Median (IQR)	0 (0-3)	1 (0-3)	0 (0-2)	0 (0-1)	

Abbreviation: MA, methamphetamine.

^a^ Recent (past 180 days) and lifetime overdose data were reported by 2854 participants.

^b^ Values were calculated with the χ^2^ test, except where noted.

^c^ Other race included Alaska Native, Asian, Pacific Islander, Native Hawaiian, and free-text responses.

^d^ Values were calculated with the Kruskal-Wallis rank sum test.

Twenty-two percent of all participants using both opioids and methamphetamine (395 of 2865 participants with nonmissing overdose data) reported experiencing an overdose in the past 180 days, compared with 14% (99 participants) of those using opioids alone and 6% (23 participants) of those using methamphetamine-alone (χ^2^_2_ = 60.7; *P* < .001). In adjusted models, co-use of methamphetamine and opioids was associated with greater odds of overdose compared with opioid use alone (adjusted odds ratio, 1.45; 95% CI, 1.08-1.94; *P* = .01) and when compared with methamphetamine alone (adjusted odds ratio, 3.26; 95% CI, 2.06-5.14; *P* < .001) (Figure). Similarly, 949 (55%) of all participants using both opioids and methamphetamine reported experiencing any lifetime overdose, compared with 293 (44%) of those using opioids alone and 110 (31%) of those using methamphetamine alone (χ^2^_2_ = 76.4; *P* < .001). People who reported using both methamphetamine and opioids reported a mean (SD) of 2.4 (4.2) (median [IQR], 1 [0-3]) lifetime overdoses vs 1.7 (3.5) (median [IQR], 0 [0-2]) among those using opioids alone (adjusted rate ratio, 1.20; 95% CI, 1.01-1.43; *P* = .04) and 1.1 (2.9) (median [IQR], 0 [0-1]) among those who used methamphetamine alone (adjusted rate ratio, 1.81; 95% CI, 1.45-2.27; *P* < .001).

**Figure.**
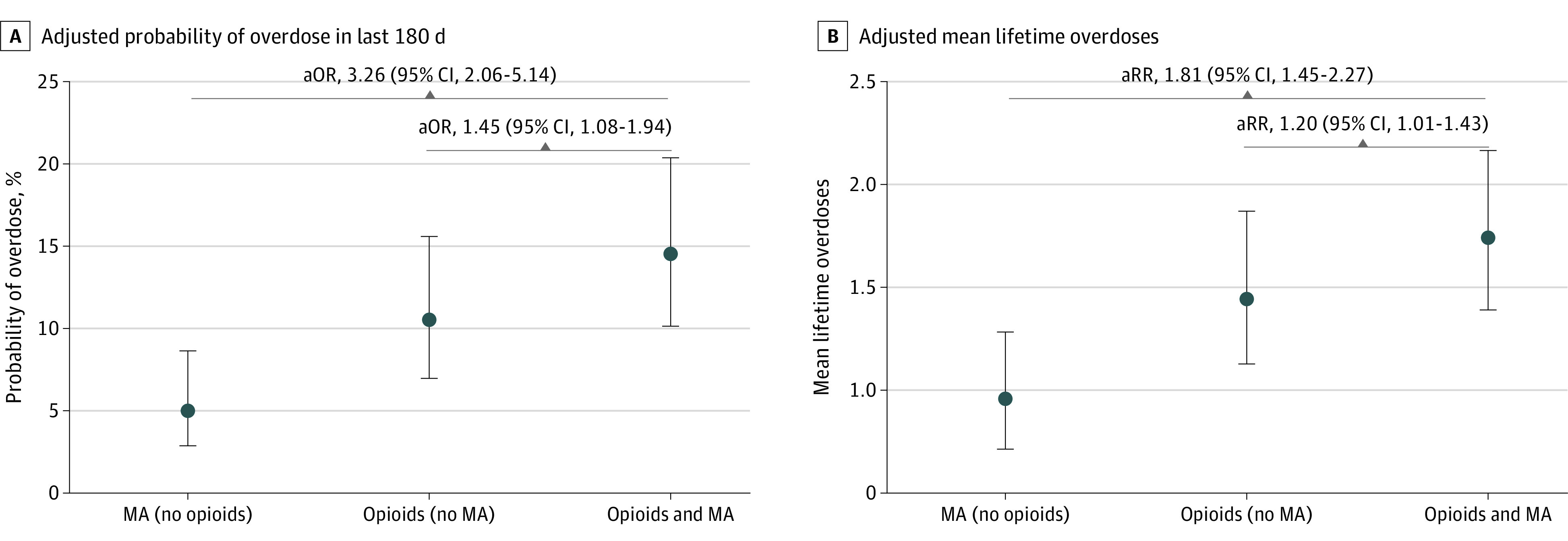
Adjusted Probability of Methamphetamine (MA) Overdose in Last 180 Days and Adjusted Mean Lifetime Overdoses Circles denote means and error bars denote 95% CIs. aOR indicates adjusted odds ratio; aRR, adjusted rate ratio.

## Discussion

The opioid overdose epidemic in rural communities was associated with pervasive methamphetamine use among people using drugs in this cross-sectional study, with approximately 4 of 5 participants reporting past-30-day methamphetamine use.^24^ Concomitant use of opioids and methamphetamine was associated with greater odds of nonfatal overdose than using opioids or methamphetamine alone. Despite this, participants with combined use reported greater difficulty accessing treatment; people using methamphetamine alone rarely had naloxone.

The high prevalence of methamphetamine use in this rural population is consistent with the National Survey on Drug Use and Health, which has documented steady increases in self-reported methamphetamine use among the general population over the past decade^6^ and increases in co-use of methamphetamine among people who use heroin,^25^ and mirrors resurgent treatment admissions for methamphetamine use disorder in the Treatment Episode Data Set data.^8^

Our geographically diverse population demonstrates the eastward march of methamphetamine use from the western US, requiring national attention. Although methamphetamine use was nearly universal among people using drugs in rural Oregon, the majority of participants in rural Wisconsin, Illinois, Kentucky, Ohio, North Carolina, and West Virginia reported recent methamphetamine use as well. This is consistent with marked increases in treatment admissions for methamphetamine use disorder in the Midwest and South, along with the western US,^8,25^ and amphetamine-related hospital admissions^11^ in the past 10 years. Even in New England, where methamphetamine prevalence was historically uncommon, nearly one-third of rural participants reported using methamphetamine.

Concomitant methamphetamine and opioid use more than tripled the odds of nonfatal overdose compared with methamphetamine use alone and increased the odds of nonfatal overdose by 39% compared with opioid use alone. Nationally, opioid overdose deaths continue to increase, driven by illicitly manufactured fentanyl^26^ with substantial coinvolvement of psychostimulants, including methamphetamine.^4,26^ As fentanyl continues to replace heroin and adulterate the methamphetamine supply,^27^ early response systems to track nonfatal overdoses and implement rapid prevention interventions are urgently needed to decrease overdose deaths. Such systems may benefit from tracking methamphetamine-related overdoses, as well as opioid-related overdoses.^28^ Harm reduction programs that distribute naloxone and fentanyl test strips to people who use methamphetamine in rural areas are needed, as most methamphetamine-related deaths also involve opioids and few people using methamphetamine reported having naloxone.^9^

The magnitude of methamphetamine use among people who use drugs has profound implications for US treatment programs. Many office-based buprenorphine providers and opioid treatment programs (ie, methadone clinics) focus on opioid treatment alone, and/or discontinue medications for opioid use disorder when methamphetamine use is identified,^29^ and express discomfort in caring for patients with polysubstance use and diversion risk.^30^ The challenge is compounded in rural communities that often lack buprenorphine prescribers: more than one-half of US counties (53.4%) do not have a buprenorphine prescriber, leaving 30 million people in those counties without access to treatment.^15^ Similarly, rural opioid treatment programs are rare, leading to long driving times to access methadone.^14^ Educational interventions are needed to train primary care practitioners—who are key providers of opioid use disorder treatment in the rural US—how to better address methamphetamine use among people who use opioids. For example, contingency management training interventions could be adapted to support rural primary care practitioners.^31,32^

People who reported using both methamphetamine and opioids also reported greater challenges in accessing substance use treatment, which is consistent with a systematic review^33^ of 39 studies that found that methamphetamine use was negatively associated with receipt of medications for opioid use disorder and retention in treatment. Our study findings suggest that access to treatment might be particularly challenging for people living in rural communities that have fewer methadone programs,^14^ buprenorphine providers,^15^ and behavioral health services,^13^ an essential component of methamphetamine use disorder treatment, for which no medications have been approved, than urban areas. Interventions to expand substance use treatment access in rural communities, such as mobile methadone delivery models^34^ and telemedicine buprenorphine interventions,^35^ should integrate evidence-based methamphetamine use disorder treatments such as contingency management.^36^

Participants with concomitant opioid and methamphetamine use were younger and more commonly reported American Indian or Alaskan Native race. Our findings in rural communities are consistent with national data that demonstrated that between 2011 and 2018, non-Hispanic American Indian or Alaskan Native individuals experienced the greatest increases and highest methamphetamine-related death rates compared with other racial and ethnic groups.^37^ Culturally tailored harm reduction and treatment interventions should be expanded for American Indian or Alaskan Native populations.

Combined opioid and methamphetamine use was more common among participants who reported recent homelessness compared with those using opioids alone. Although people experiencing homelessness often use methamphetamine to mitigate the challenges of poverty, combined use of methamphetamine and opioids may also indicate substance use severity that increases housing instability. Other markers of increased addiction severity among those with combined methamphetamine and opioid use included greater daily drug injection and binge drinking, suggesting the need for more intensive treatment for people with multiple substance use disorders.

### Limitations

Our study should be interpreted in view of several potential limitations. First, the data are cross-sectional, which limits inferences of causality. Longitudinal cohorts of people who use drugs in rural communities are needed to better characterize the dynamic nature of substance use and strengthen capacity for causal inference. Second, although the ROI constitutes the largest assembled cohort of people who use drugs in rural America and RDS weights may allow generalizability to the participating communities, it is not a national probability sample and likely underrepresents important segments of the rural population. Third, substance use data were self-reported; the true prevalence of methamphetamine use in rural communities may be greater. Fourth, fentanyl adulteration of both methamphetamine and heroin supplies may have created some misclassification bias, possibly leading to underestimation of true associations with overdose. Fifth, our models of lifetime overdose counts were unable to adjust for length of individuals’ time at risk for overdose, as the ROI did not collect data on participants’ age at first drug use. Our models also assume that drug use patterns were constant over time at risk, which is unlikely to be true, but we lack the data to verify or adjust for this. Sixth, the recall times for overdose exceeded that of past-30-day drug use, allowing for outcomes that may have preceded the exposure. Seventh, the ROI did not collect overdose death data. Future cohort development should consider linking participants to national vital statistics data.

## Conclusions

Our results document pervasive co-use of methamphetamine and opioids associated with nonfatal overdose among people who use drugs in rural communities, with profound implications for harm reduction and treatment services. Study findings highlight the urgent need for interventions that address both opioids and methamphetamine and are tailored to the needs of rural communities.

## Notes

Supplement. **eTable 1.** Participant Survey Items for Eligibility and Measures **eTable 2.** Overall and Site-Specific Prevalence of Methamphetamine (MA) Use, N = 1271 Rural Opioid Initiative Participants at an RDS Depth of 3 or Higher
