# Supplementary material for: Association of Methamphetamine and Opioid Use With Nonfatal Overdose in Rural Communities
Source: JAMA Netw Open. 2022 Aug 15;5(8):e2226544. doi: 10.1001/jamanetworkopen.2022.26544 (PMC9379740; doi:10.1001/jamanetworkopen.2022.26544)
Supplement: Supplement. — eTable 1. Participant Survey Items for Eligibility and Measures eTable 2. Overall and Site-Specific Prevalence of Methamphetamine (MA) Use, N = 1271 Rural Opioid Initiative Participants at an RDS Depth of 3 or Higher [file jamanetwopen-e2226544-s001.pdf]

Supplemental Online Content

Korthuis PT, Cook RR, Foot CA, et al. Association of methamphetamine and opioid use with nonfatal overdose in rural communities. *JAMA Netw Open*. 2022;5(8):e2226544. doi:10.1001/jamanetworkopen.2022.26544

eTable 1. Participant Survey Items for Eligibility and Measures

eTable 2. Overall and Site-Specific Prevalence of Methamphetamine (MA) Use, N = 1271 Rural Opioid Initiative Participants at an RDS Depth of 3 or Higher

This supplemental material has been provided by the authors to give readers additional information about their work.

© 2022 Korthuis PT et al. *JAMA Network Open.*

eTable 1. Participant Survey Items for Eligibility and Measures

Purpose	Questions and Response Options		
Background			
Eligibility	How old are you?		________ *years old*
Eligibility	What is the zip code where you have slept most in the past 30 days?<br>(If you don't know, ask the research staff person for help.)		___ ___ ___ ___ ___
**Drug Use**<br>Have you ever used ______to get high?			
Eligibility, Measure	… heroin?	☐ Yes<br>☐ No<br>☐ Don't know	If yes, when did you last use heroin to get high? Please enter the two digit MONTH, followed by the two digit DAY, followed by the four digit YEAR. It's ok if you don't remember the exact date. Your best guess is fine.<br><br>___/ ____/________<br>MO/DAY/YEAR
Eligibility, Measure	... street fentanyl or carfentanil powder?	☐ Yes<br>☐ No<br>☐ Don't know	If yes, when did you last use fentanyl or carfentanil to get high? Please enter the two digit MONTH, followed by the two digit DAY, followed by the four digit YEAR. It's ok if you don't remember the exact date. Your best guess is fine.<br><br>___/ ____/______<br>MO/DAY/YEAR
Eligibility, Measure	...opiate painkillers – like oxycodone, Percocet, Percodan, OxyContin, hydrocodone, Vicodin, Lorcet, Lortab, Norco, Morphine, Dilaudid, Opana, T3s, fentanyl patch etc.?	☐ Yes<br>☐ No<br>☐ Don't know	If yes, when did you last use opiate painkillers to get high? Please enter the two digit MONTH, followed by the two digit DAY, followed by the four digit YEAR. It's ok if you don't remember the exact date. Your best guess is fine.<br><br>___/ ____/________<br>MO/DAY/YEAR
Measure	...methamphetamine, crystal meth or amphetamine?	☐ Yes<br>☐ No<br>☐ Don't know	If yes, when did you last use methamphetamine, crystal meth or amphetamine to get high? Please enter the two digit MONTH, followed by the two digit DAY, followed by the four digit YEAR. It's ok if you don't remember the exact date. Your best guess is fine.<br><br>___/ ___/______<br>MO/DAY/YEAR

© 2022 Korthuis PT et al. *JAMA Network Open.*

Eligibility, Measure	Have you ever **<u>injected</u>** drugs to get high?	☐ Yes<br>☐ No	If yes, when did you last **<u>inject</u>** drugs to get high? Please enter the two digit MONTH, followed by the two digit DAY, followed by the four digit YEAR. It's OK if you don't remember the exact date. Your best guess is fine.<br><br>___/ ___/_______<br>MO/DAY/YEAR
**Overdose Experiences**			
Measure	Have you ever overdosed? By overdose, I mean if you passed out, turned blue, or stopped breathing from using drugs.	☐ Yes<br>☐ No<br>☐ Don't know	
Measure	How many times in your life have you overdosed?	☐ ____________ times	
Measure	When was the last time you had an overdose?<br>Please enter the two digit MONTH followed by the two digit DAY followed by the four digit YEAR. It's OK if you don't remember the exact date. Your best guess is fine.	_________ / _________ /_________<br>Month Day Year	
**Treatment Access**			
Measure	In the past 6 months, did you try to get any of the following treatments but were unable to?<br><br>Choose all that apply.	☐ Buprenorphine maintenance – like Suboxone or Subutex – from a doctor or program<br>☐ Methadone maintenance from a clinic<br>☐ Naltrexone shots – like Vivitrol<br>☐ Buprenorphine shots – like Sublocade<br>☐ Outpatient drug treatment<br>☐ Residential or inpatient drug treatment<br>☐ Drug detox	
**Naloxone Possession**			
Measure	Do you currently have naloxone or Narcan with you or at home?	☐ Yes<br>☐ No<br>☐ Don't know	

© 2022 Korthuis PT et al. *JAMA Network Open.*

eTable 2. Overall and Site-Specific Prevalence of Methamphetamine (MA) Use, N = 1271 Rural Opioid Initiative Participants at an RDS Depth of 3 or Higher.

	Unweighted		Weighted	
Region	N (%)	MA Prevalence % (95% CI)	N (%)	MA Prevalence % (95% CI)
Overall	1271 (100)	86 (70, 94)	4,484 (100)	83 (65, 93)
IL	38 (3)	82 (68, 92)	117 (3)	89 (76, 97)
KY	208 (16)	82 (76, 87)	911 (20)	67 (52, 82)
NC	181 (14)	92 (88, 96)	641 (14)	90 (83, 96)
NE[a]	372 (29)	33 (28, 38)	544 (12)	31 (26, 36)
OH	129 (10)	89 (84, 94)	397 (9)	78 (61, 93)
OR	57 (4)	98 (95, 100)	174 (4)	97 (90, 100)
WI	286 (23)	90 (86, 93)	1,702 (38)	88 (82, 93)
WV	NA	NA	NA	NA

[a]Includes Massachusetts, New Hampshire, Vermont.

MA = methamphetamine. CI = Confidence Interval. NA = Not applicable; West Virginia did not collect respondent driven sampling data required for calculating depth.

© 2022 Korthuis PT et al. *JAMA Network Open.*
